# How to Compute Density Fluctuations at the Nanoscale

**DOI:** 10.1021/acs.jctc.4c01047

**Published:** 2024-12-27

**Authors:** Peter Krüger

**Affiliations:** Materials Science Department, Graduate School of Engineering, Chiba University, Chiba 263-8522, Japan

## Abstract

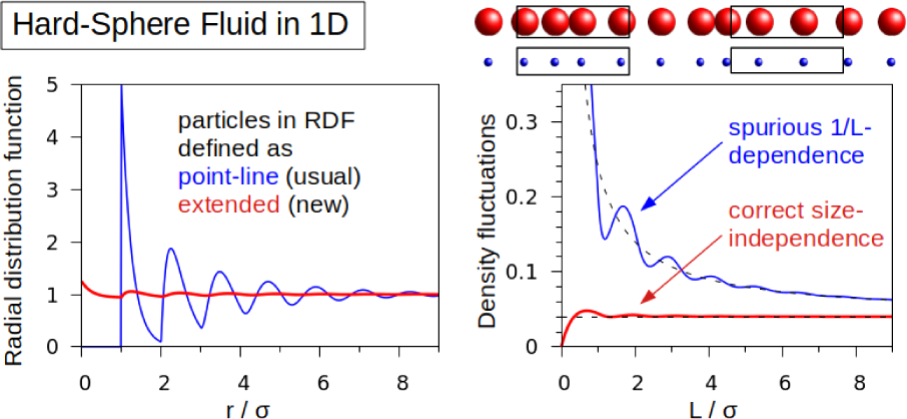

The standard definition of particle number fluctuations based on point-like particles neglects the excluded volume effect. This leads to a large and systematic finite-size scaling and an unphysical surface term in the isothermal compressibility. We correct these errors by introducing a modified pair distribution function that takes account of the finite size of the particles. For the hard sphere fluid in one-dimension, we show that the compressibility is strictly size-independent, and we reproduce this result from the number fluctuations calculated with the new theory. In general, the present method eliminates the leading finite-size effect, which makes it possible to compute density fluctuations accurately in very small sampling volumes, comparable to a single particle size. These findings open the way for obtaining the local compressibility from fluctuation theory at the nanometer scale.

## 1 Introduction

From fundamental principles of statistical mechanics, response functions are directly linked to the fluctuations of some extensive variable, e.g., the specific heat is related to energy fluctuations and the compressibility to volume or number fluctuations.^1^ In inhomogeneous matter, intensive variables, including response functions, are position-dependent. This raises the question of whether fluctuation theory can still be used at length scales comparable to the particle size.

Various methods have been proposed for generalizing thermodynamics to systems at the nanometer scale,^2−9^ many of which are based on Hill’s thermodynamics of small systems.^3^ Hill considered statistical ensembles of finite-sized systems, such as droplets and clusters. Here, we focus on the related but somewhat different problem of a finite, open subvolume inside a large system. We ask the question whether the response functions can be accurately calculated from the knowledge of the fluctuations inside the subvolume alone. This is a crucial issue for confined or otherwise inhomogeneous systems, where the local intensive variables, including the response functions, may vary in space on the nanometer scale. For example, the spatial variation of the solvent density fluctuations around a solute molecule has been shown to give important insights into the thermodynamics of solvation^10^ and about drying and wetting phenomena at surfaces and large solute particles.^11,12^ In order to discuss such systems using thermodynamic language, the response functions of a small open subvolume must be unambiguously defined and computed in an efficient way.

Building on Hill’s approach, a theory of nanothermodynamics has been formulated over the last two decades by the Trondheim group.^13^ In their small system method, response functions are obtained from the fluctuations inside a finite subvolume embedded in a large reservoir.^5^ It was found that in homogeneous fluids, the particle number fluctuations and the isothermal compressibility are strongly size-dependent at the nanometer scale.^14^ For example, the compressibility of liquid water in a subvolume of size 1 nm^3^ was found to be three times larger than the bulk compressibility. This was attributed to a large surface term.

Here, we argue that such a large surface term in the compressibility of a fluid is a numerical artifact induced by the discreteness of the particle number operator. We show that the surface term essentially vanishes when the excluded volume effect is taken into account in the definition of the density fluctuations. The importance of the excluded volume for the finite-size effects of the X-ray scattering factor has been stressed recently.^15^ Here, we demonstrate that the isothermal compressibility of a hard sphere fluid (HSF) in one dimension (1D) is size-independent down to the smallest physically meaningful size of a single particle. We show that this result can also be obtained from the particle number fluctuations in an open subvolume if the compressibility equation is corrected in two ways. First, a modified radial distribution function (RDF) is introduced, which takes account of the finite particle size, i.e., the excluded volume effect. The modified RDF can be obtained from the usual RDF by simple convolution. Second, the compressibility equation must be evaluated using the finite-volume Kirkwood–Buff integral (KBI) theory^16−20^ rather than the standard running KBI.^21,22^ For the 1D HSF at high filling, we find that the fluctuations obtained with the modified RDF are virtually size-independent down to subvolumes containing a single particle. At low filling, some size-dependence remains, but it can be made to vanish by using a reduced particle size in the modified RDF. By demanding that the compressibility be exactly size-independent, which holds for the 1D HSF in the canonical and the isobaric ensemble, we define an effective particle diameter that agrees with the hard-sphere diameter at large filling. We also apply the method to the HSF in 3D using the RDF in the Percus–Yevick approximation^23^ and to the hard sphere solid, and we obtain qualitatively the same results as in 1D. Our findings show that density fluctuations can be defined unambiguously at the nanometer scale and that the local compressibility can be computed accurately with sampling volumes of minimum size. We note, however, that for response functions, which are directly related to the discrete particle *number* fluctuations rather than the continuous *density* fluctuations, such as the chemical potential derivatives^5^ and excess free energies in solvation theory,^24^ the present method might not be suitable, and the usual definition of the RDF with point-like particles^22^ should be kept.

## 2 The Isothermal Compressibility from Density Fluctuations in Different Ensembles

The isothermal compressibility of a piece of matter held at temperature *T* and pressure *p* and occupying a volume *V* is a mechanical response function, defined as . For a system containing *N* identical particles, the thermodynamic definition becomes
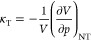
1From fluctuation theory, we have
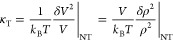
2where  is the particle number density. We write  for the average and  for the fluctuation of a quantity *A*. In eq 2, the statistical averages are taken in the isothermal–isobaric (*NpT*) ensemble. In fluids, it is often more convenient to work in the grand-canonical () ensemble, and the following definition is commonly used, which relates the compressibility to the particle number fluctuations in an open volume *V*
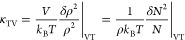
3Clearly, the only difference between eqs 2 and 3 is the statistical ensemble, which is indicated in κ_TV_ by the extra subscript V. For a macroscopic system, all ensembles are equivalent and so . For small systems, there is no general equivalence between ensembles. Equation 2 is valid on general statistical mechanical grounds for any number of particles *N* > 0. The only condition is that the *reservoir* is large enough that the volume fluctuations are approximately Gaussian. For the HSF in 1D, we shall prove explicitly that eq 2 holds for any *N*. However, we find , and thus eq 3 should not be used to calculate the compressibility of small systems. The reason why  can be understood as follows. The compressibility κ_T_ is obviously a continuous variable, as are all the quantities used in eqs 1 and 2. In contrast, the particle number *N*, whose fluctuations are used in eq 3 is a discrete variable. For nanosystems,  is of the order of 1, and the probability distribution of *N* is not at all Gaussian. As a consequence,  differs from . However, eq 3 is more convenient for fluids than eq 2 because eq 3 corresponds to an open volume in an  ensemble. Using a finite open volume, one can, in principle, study the compressibility, density, and concentration fluctuations *locally* in inhomogeneous or confined fluids.^13^ This is not possible with eq 2, which corresponds to a closed system.

The fact that *N* is a discrete variable is related to the assumption of point-like particles, which is implicit in the definition of the *n*-particle densities. The 1-particle density is
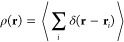
4where the brackets denote the ensemble average and *i* counts the particles. We define the 2-particle density as
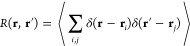
5Note that the diagonal terms *i* = *j* are included in the sum. In the more common definition,^22^ denoted ρ^(2)^, the diagonal terms are omitted. The two definitions are equivalent, and we have . We use *R* because it is a more suitable starting point for the density fluctuations for particles of finite size, which will be introduced below. According to eq 4, the *instantaneous* particle density for a single microstate diverges at the particle positions . While this may approximately be true for the mass density in a gas of atoms, it is not a good model for the present purpose because it does not account for the size of the particles, which may be atoms or molecules. Instead of the mass density, it is better to think of the electronic density, which determines the size of the particles and explains the excluded volume effect by Pauli repulsion. If the individual particles are given a finite size and density, then *N* becomes a continuous variable because a particle near the boundary of an open volume *V* can be partially inside and partially outside of *V*. In Section 4, we shall introduce modified particle densities, which take into account the finite size of the particles. We shall show that the error of κ_TV_ can thereby be corrected for.

The particle number fluctuations  in an open volume *V* are given by
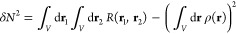
6In the following, we focus on a homogeneous and isotropic fluid, where ρ is a constant, and *R* only depends on the distance . Then, eq 6 can be simplified to^16^
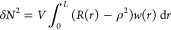
7where *w*(*r*) is a geometrical weight function that depends only on the size and shape of the volume *V*,^16,18^ and *L* is the maximum distance in *V*. For a 1D system of length *L*, we have , and for a 3D sphere of diameter *L*, we have . Equation 7 can be written in terms of the RDF, which is given by
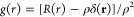
8With , we obtain, from eqs 7 and 8 for the number fluctuations per particle^16^
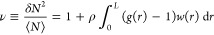
9

## 3 Hard Sphere Fluid in 1D

**Figure 1:**
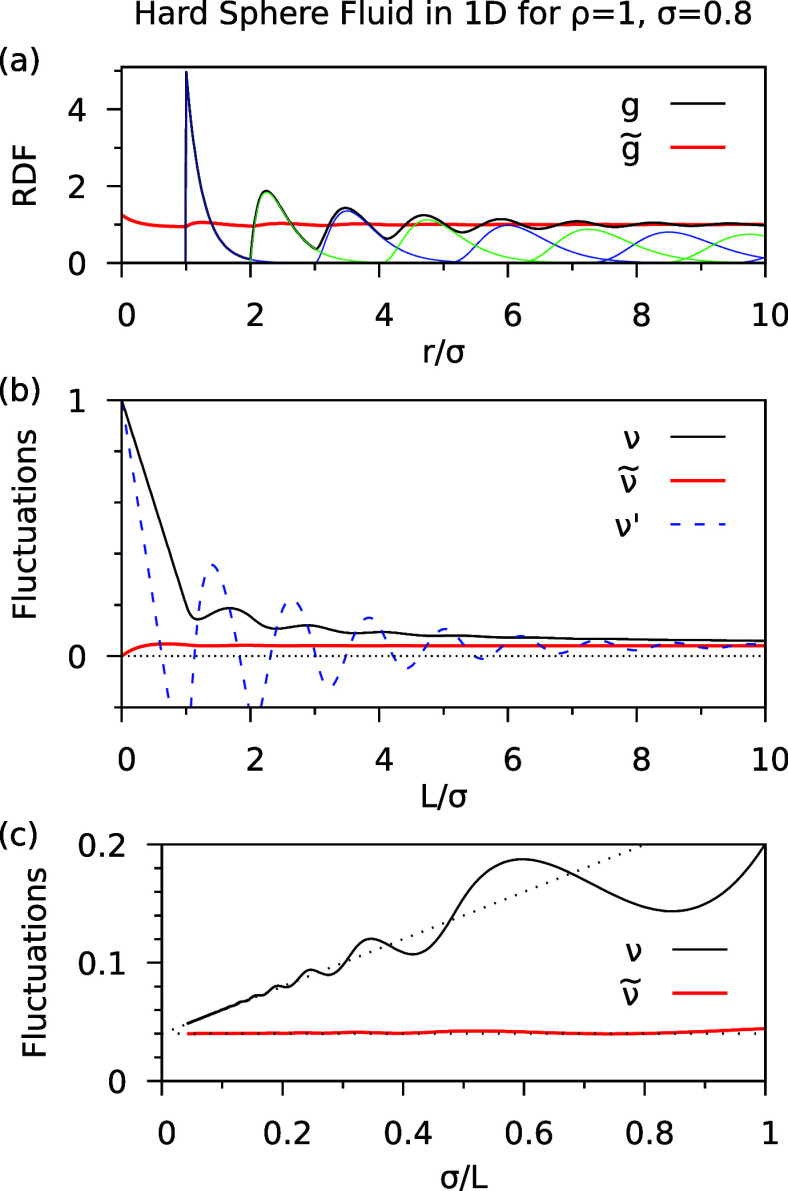
Results for hard sphere fluid in 1D with ρ = 1, . (a) Usual RDF *g*(*r*) (black line) with components , eq 12 for *n* even (green) and odd (blue). Modified RDF  (red). (b) Relative number fluctuations ν and  () as a function of finite system size *L*.  (dashed blue) is obtained with *g*(*r*) and the “Running-KBI”, i.e., by putting  in eq 9. (c) ν and  as a function of . The thin black lines are linear interpolations, namely ν = 0.04 and .

We first consider the hard sphere fluid (HSF) in 1D, also known as the Tonks model,^25^ and we study the size dependence of the isothermal compressibility and the density fluctuations. In 1D, force *F* and pressure *p* are the same. The system length *L* is the 1D hypervolume *V*. With these notations, the exact equation of the state of the Tonks model^25^ with *N* particles of diameter σ is given by . In the canonical ensemble, this equation of state is valid for any number of particles *N* and any system size . The isothermal compressibility follows directly from the definition, eq 2, and we obtain
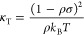
10Note that the compressibility (eq 10) is clearly size-independent. Next, we calculate κ_T_ from the fluctuation theory. First, we consider volume fluctuations in the *NpT* ensemble. To this end, we employ the grand-canonical RDF of the Tonks model, which is known analytically^26^
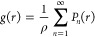
11where
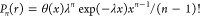
12Here, we have put , , and  is the Heaviside step function.  is the probability that the distance between a given particle and its *n*th nearest neighbor to the right lies in . The RDF *g*(*r*) and its components  are plotted in Figure 1a for a HSF in 1D with ρ = 1, σ = 0.8.

In the 1D HSF, the distance distribution  can also be interpreted as the length distribution of a closed system of *N* particles in an *NpT* ensemble. This was explicitly shown by Tonks^25^ for *N* = 1, and the argument can easily be generalized to *N* > 1. It follows that the expectation value and the variance of the system length (*L*) in the *NpT* ensemble can be obtained from the first two moments of the  distribution, i.e., eq 12 with *n* = *N*, *r* = *L*. The *k*th moment is . The calculation is straightforward and gives
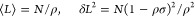
13These expressions are valid for any *N*, i.e., for any system size in the *NpT* ensemble. Upon identifying  with , we obtain, from eq 2, exactly the same expression for κ_T_ as from the thermodynamic definition eq 10. Thus, we have demonstrated that for the 1D HSF, the hyper-volume fluctuations in the *NpT* ensemble give the exact, size-independent, isothermal compressibility.

Next, we study the density fluctuations of the 1D HSF in the grand-canonical ensemble, on an open segment of finite length . We have  and  can be calculated from eqs 9, 11, and 12. The relative fluctuations  are shown in Figure 1b,c (black lines). ν has a pronounced size dependence and becomes linear in  for large *L*. For , ν goes to  (=0.04 in this case). This is the exact limit, which follows from eq 10 and the fact that  in the thermodynamic limit. Since the exact isothermal compressibility, κ_T_, eq 10, is strictly size-independent, it is clear that the large size dependence of ν (and thus κ_TV_) seen in Figure 1b,c, is a nonphysical result. We conclude that the particle number fluctuations (ν defined in the usual way with eqs 5 and 6 cannot be used to compute the isothermal compressibility for finite-size systems. For completeness, we also show the result obtained when using the standard or “running”-KBI instead of the finite-volume KBI (eq 9). For the running KBI, the infinite-volume expression is simply truncated at the upper bound *L*. It can be computed from eq 9 upon replacing *w*(*r*) by 2 in 1D or  in 3D. The number fluctuations of the 1D HSF, computed with the running KBI are plotted in Figure 1b as  (blue dashed line). The function  oscillates strongly and thus has an even larger size dependence than ν. Most importantly,  becomes negative for certain system sizes *L*, which is obviously wrong, since fluctuations cannot be negative on mathematical grounds. This means that running-KBI cannot be used to assess finite-volume density fluctuations.

## 4 Modified RDF with Excluded Volume

We have seen above that the difference between κ_TV_ and κ_T_ is related to the discreteness of the particle number and we have shown that the usual, point-like definition of the particle densities leads to unphysical results for the compressibility κ_TV_ of small systems. Now, we go beyond the point-like particle picture and take account of the fact that real particles have a finite size, by replacing the  functions in the definition of the *n*-particle densities, by a regular function , which describes the finite density of the individual particles. Here, we consider hyper-spherical particles with diameter *a* and constant internal density,
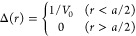
14where *V*_0_ is the hyper volume, i.e.,  in 1D and  in 3D. Such a step-like density profile with  is the natural choice for the hard-sphere model. However, other simple density profiles lead to very similar results and to the same conclusions, as we shall show explicitly for a Gaussian profile in a forthcoming paper.

By attributing a finite size to the particles in the modified *n*-particle densities, the number of particles *N* in a small volume becomes a continuous variable rather than a discrete variable. However, the underlying statistical mechanical calculations are not changed in any way, and the discreteness of the particle number as a physical observable is respected. The variable *N* is taken as continuous only when considering the particle density fluctuations in a finite open volume from the microscopic states. The modified quantities, obtained upon replacing  by , are denoted by , ,  etc. Introducing a spherically symmetric internal density  does not break the symmetry of the fluid. As a consequence, the modified 1-particle density is unchanged,  and the modified 2-particle density, given by
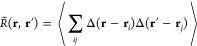
15only depends on the particle distance . The function  can be obtained from *R*(*r*) by a convolution
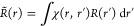
16as we have shown previously.^27^ Note that in ref. (27), the convolution was derived for  from *g*(*r*), where the diagonal terms (*i* = *j*) are omitted, but this has no effect on the convolution, i.e., the functions  for  are the same as those for  in ref (27). It is easy to see that in 1D, we have  for  and χ = 0 otherwise.

The particle number fluctuations can be computed in the same way as before upon replacing *R*(*r*) by  in eq 7. We define the modified RDF  as
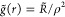
17In this definition, the diagonal (*i* = *j*) terms in the two-point density eq 15 are included. In the definition of the usual RDF *g*(*r*), the diagonal terms are omitted because they would lead to a delta-function at *r* = 0. With the modified RDF, the relative fluctuations are given by
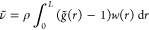
18Note that the constant 1 in the expression for ν, eq 9, which compensates for the omitted diagonal terms in *g*(*r*), is absent in eq 18.

For the 1D HSF with , the modified RDF  is shown in Figure 1a as the red line. The large oscillations of *g*(*r*) are almost completely suppressed and  for all . The corresponding particle number fluctuations  in an open system of length *L* are shown as red lines in Figure 1b,c. Except of ,  is almost constant at 0.04, which is the correct value in the thermodynamic limit. This means that the density fluctuations are virtually size-independent down to the smallest physically meaningful system size of one particle () in agreement with the analysis in the *NpT* ensemble. Importantly, it follows that , i.e., the true compressibility κ_T_ can be approximated, with very good accuracy by computing κ_VT_ from the particle number fluctuations in an open volume *V* of minimum size, provided that the modified RDF is used.

**Figure 2:**
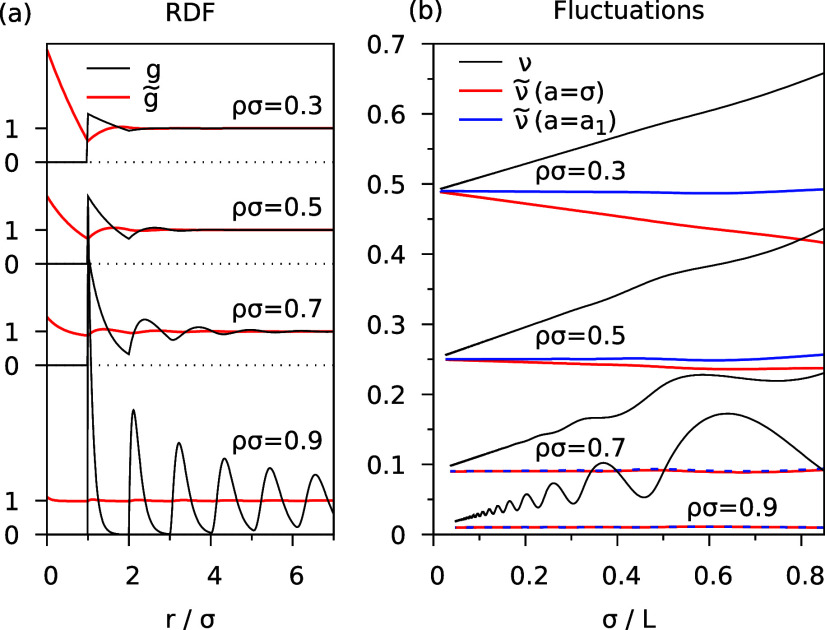
Results of the hard sphere fluid in 1*D* as a function of filling fraction ρσ. (a) Usual RDF (*g*) and modified RDF () for . (b) Corresponding number fluctuations.

In Figure 1, we have considered a large filling fraction , appropriate for modeling a liquid. The dependence of the RDF and the fluctuations on the density ρ is shown in Figure 2a,b. For decreasing ρ, the oscillations of the usual RDF *g*(*r*) become smaller, and *g*(*r*) approaches a step function for ρ → 0. At the same time, the modified RDF  deviates more strongly from 1 for small *r* up to about . The peak seen at *r* = 0 (for small ρ) is due to the diagonal (*i* = *j*) terms in eq 15. It evolves to a delta-function in the limit .

Looking at Figure 2b, we see that in all cases, the relative fluctuations tend to the exact result  in the limit . The functions  (black lines) which were obtained with the usual RDF *g*(*r*), show a strong size dependence that is linear in  for large *L*. Interestingly, the slope, which may be interpreted as a surface contribution,^14,28^ hardly depends on the density ρ (at constant σ) but scales linearly with σ (at constant ρ). This suggests that the surface contribution to ν is essentially determined by the size of the individual particles or their excluded volume, rather than by long-range correlations in the fluid.

The fluctuations obtained with the modified RDF are shown in Figure 2b for two choices of the diameter *a* in eq 14, namely the “natural” choice  and a “corrected” value  defined below. Considering first  (red lines), we see that for high density (, typical for a liquid phase) the fluctuations  are virtually independent of *L*. However, for lower densities () a non-negligible size dependence appears, and  deviates from  by a negative term which is linear in . We recall that the aim of the RDF modification is to obtain density fluctuations in finite open systems that agree with those in the *NpT* ensemble and which thus yield the physically correct compressibility, i.e., . Since the κ_T_ is strictly size-independent in the 1D HSF, we choose the parameter *a* in eq 14 such that the size-dependence of the number fluctuations  becomes as small as possible. Empirically, we find that , where
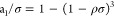
19gives excellent results for all densities, as seen in Figure 2b, blue lines. Note that the difference between *a*_1_ and the hard-sphere diameter σ is negligible for high density, e.g., for , it is merely 0.8%. In the low-density limit, ρ→ 0, we have , which means that the modified RDF goes back to the usual, point-like definition. This is consistent with the fact that in the zero density limit, the HSF behaves like an ideal gas.

The need for reducing the particle size may seem to introduce some empirical element into the theory. This is not the case, however, since the reduction factor is determined by physical conditions. Indeed, our aim is to define the number fluctuations in a small volume in such a way as to obtain the same density fluctuations as in the isobaric ensemble, or equivalently, we demand  in eqs 2and 3^3^). Since κ_T_ is size-independent as shown above, so must be κ_TV_ and , which is the condition we use for the particle size reduction. In the Appendix, we show that if the compressibility of a small open subsystem of a homogeneous fluid depends on the subsystem size, then contradictions with general thermodynamic principles arise. A physically meaningful definition of the local compressibility is size-independent. As a consequence, if the compressibility is to be obtained from fluctuation theory, then the local density fluctuations in a small open system should be free of any systematic size-dependence. We note, however, that the density fluctuations are strictly size-independent only for the hard-sphere fluid. In systems with attractive interactions, the particle number fluctuations may display a significant size dependence, which is related to the nucleation of a liquid–vapor interface.^29^ Application of the present formalism to such cases will be reported elsewhere. The new method will remove the spurious  dependence due to the neglect of the excluded volume effect, but can be expected to leave the physical size dependence qualitatively unchanged.

## 5 Hard Sphere Fluid in 3*D*

**Figure 3:**
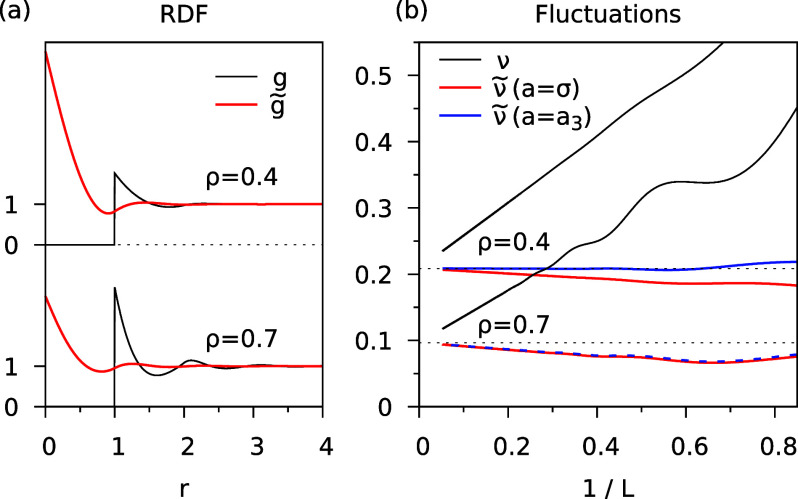
Hard sphere fluid in 3*D* with σ = 1 and different densities ρ. (a) Usual RDF (*g*) and modified RDF () for . (b) Corresponding number fluctuations.

For the HSF in 3D, exact analytic solutions are not known. Here, we use the solution of the Ornstein–Zernike integral equations in the Percus–Yevick approximation (PYA).^23^ The corresponding RDF was given by Wertheim,^30^ and we use the implementation by Kelly et al.^31^ In the 3D HSF, the density  is related to the filling fraction η as . We put σ = 1 in the following. In Figure 3a, the RDF *g*(*r*) is shown for ρ = 0.4 and ρ = 0.7. As in 1D, the modified RDF  can be obtained by a convolution, eq 16. In 3D, for the constant density profile eq 14, the kernel function χ is given by^27^
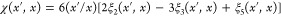
20where  and
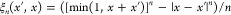
21This holds for  while χ = 0 for . The modified RDF  with  is shown in Figure 3a. Compared to *g*(*r*), the oscillations are strongly suppressed in , which is very flat and close to 1 for all *r* > 1.5.

We note that for nonspherical particles, the hard-sphere density profile of eq 14 is not suitable. It should be replaced by a profile obtained by averaging over all particle orientations. As a consequence, the kernel function  will in general not have any simple analytic form, but the one-dimensional convolution, eq 16, is still valid, and the numerical calculation will not become more difficult.

The relative number fluctuations  are plotted in Figure 3b as a function of , where *L* is the diameter of the spherical subvolume *V*. The values ν, obtained with the usual RDF *g*(*r*), show a  scaling with a large slope. For ρ = 0.7, the value of ν at , which corresponds to a subvolume containing  particles, is about twice as large as the bulk value . This finding is in qualitative agreement with the study on liquid water by Strom et al.^14^

The results of a linear fit  in the region  are given in Table 1, also for other densities not shown in Figure 3. We also listed the values of ν obtained from the equation of state (EoS) by Carnahan and Starling.^32^ The EoS is given by  where  is the filling fraction. The isothermal compressibility is readily obtained from the definition, eq 1, and together with , we have . Despite its simplicity, the Carnahan–Starling EoS is known to be very reliable for low and moderate densities up to about ρ = 0.9.^33^

**Table 1:** Relative Number Fluctuations ν of the 3D HSF as a Function of Density ρ^a^)

ρ	*ν*(∞)	*ν̃*(∞)	ν(EoS)	rel.err	*C*	*C̃*	*a*_3_/*σ*
0.1	0.6635	0.6635	0.6611	0.003	0.318	0.001	0.429
0.2	0.4448	0.4449	0.4406	0.010	0.454	0.000	0.674
0.3	0.3017	0.3019	0.2949	0.023	0.499	0.001	0.814
0.4	0.2086	0.2088	0.1974	0.057	0.498	–0.002	0.894
0.5	0.1494	0.1495	0.1317	0.134	0.473	–0.011	0.939
0.6	0.1141	0.1141	0.0872	0.308	0.438	–0.027	0.965
0.7	0.0964	0.0965	0.0570	0.691	0.397	–0.049	0.980

a Result of the linear fits ν(*L*) = ν(∞) + *C*/*L* and ν̃(*L*) = ν̃(∞) + *C̃*/*L* (for *a* = *a*_3_). ν(EoS) corresponds to the Carnahan–Starling equation of state. The relative error (“rel.err”) is defined as ν(∞)/ν(EoS)–1. The values *a*_3_/σ were used for ν̃ and correspond to eq 22

From Table 1, we see that the  values obtained with the RDF in PYA agree very well with the EoS for low density, with an error of a few percent for . For higher density, the error rises quickly and reaches 70% for ρ = 0.7. Therefore, the results obtained for ρ > 0.4 must be considered preliminary due to the approximate nature of the RDF in PYA.

In Figure 3b, the fluctuations , obtained with the modified RDF  with  (red lines) are roughly constant for , in stark contrast to the large size dependence of . In order to make  as size-independent as possible, we use a reduced particle size in the calculation of the modified RDF. In 3D, we put , where
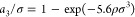
22The numerical values  are given in the last column of Table 1. This empirical correction works very well for .  is virtually size-independent, as seen directly from Figure 3b and from the negligible values of the slope . For ρ > 0.5, the  values are not negligible, and some -like size dependence remains. We do not attempt to correct for this by adjusting *a*_3_, because we attribute the residual size dependence to the quite large error of  (see Table 1) coming from the RDF in PYA, rather than to the functional form of *a*_3_.

In Table 1, the values  and  agree almost perfectly. Let us note that theoretically,  holds whatever the choice of *a*. Indeed, it is easy to see from the definition 15 that attributing a finite density to the particles does not change the relative number fluctuations in the thermodynamic limit.^27^

In this paper, we have focused on systems with a single species. In the case of mixtures, each species *i* will have a different particle size. The best choice for the effective particle sizes *a*_*i*_ is not obvious and needs to be studied in detail. A simple model consists in fixing the ratios  to the corresponding ratios of the free particle sizes and adjusting the remaining overall parameter so as to minimize the size dependence of the compressibility, as was done here for the single species case.

**Figure 4:**
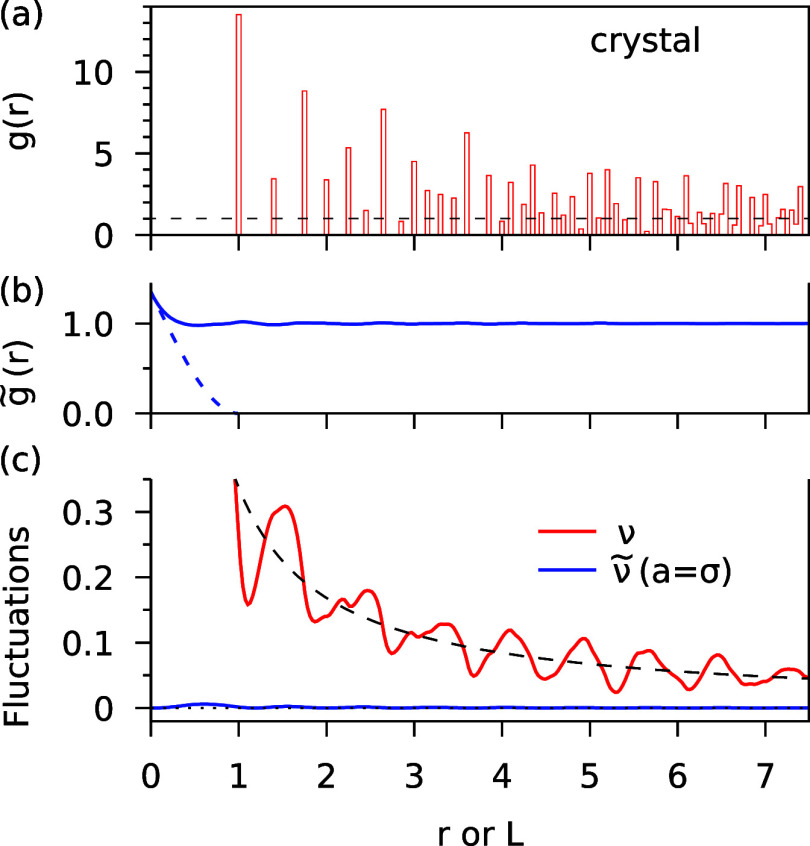
(a) RDF *g*(*r*) of a perfect face-centered cubic crystal The delta functions are replaced by bars of finite width 0.05. (b) Modified RDF  with . The dashed line is the contribution from the diagonal (*i* = *j*) terms in eq 15. (c) Relative fluctuations  (red) and  (blue). We have  and . The thin dotted line is a guide to the eye given by .

Finally, we apply our method to a hard sphere solid at zero temperature. It has a face-centered cubic crystal structure and the largest possible filling fraction of . The density is . Since the atoms are immobile, the usual RDF is a sum of delta functions, shown as a histogram in Figure 4a for σ = 1. The modified RDF  with  is the blue solid line in Figure 4b. The blue dashed line is the contribution to  from the terms *i* = *j* in the two-particle density, eq 15. These terms are omitted in *g*(*r*), since they correspond to a term , which integrates to 1 in ν in any system. For particles with finite density, however, the diagonal terms are nontrivial and their integral leads to a nonsingular peak in  near *r* = 0 (dashed line in Figure 4b). This peak fills the “hole” of the RDF for , which is present in all real systems due to the excluded volume effect. Quite surprisingly,  is virtually constant and equal to 1 for all *r* and thus very similar to the RDF of the ideal gas (). The crucial difference comes from the diagonal terms of the two-particle density that are included in  but not in *g*. In the ideal gas, these terms lead to ν = 1, while in  of the solid, they just fill the hole of the excluded volume, so that  as seen in Figure 4c.

The fluctuations are shown in Figure 4c. The function  has large oscillations, which are due to the long-range correlations in *g*(*r*). It shows the familiar  scaling and converges to the correct thermodynamic limit .^34^ Note that this trivial result cannot be obtained from the standard expression of the compressibility equation,^22^ i.e., with  instead of *w*(*r*) in eq 9. Indeed, the running-KBI strongly diverges in solids, and the finite-volume KBI theory must be used to obtain convergent results.^27,34^ While  is correct,  for finite *L* is not. In a perfect crystal at *T* = 0 K, all atoms are immobile, and so the density fluctuations and the compressibility are strictly zero, whatever the size of the system. In other words, the whole functional form of  (i.e., its deviation from zero) is a finite-size effect and has no physical meaning. In stark contrast to , the function  obtained with the modified RDF is very nearly constant and equal to zero for any *L*, which is the physically correct result.

In this paper, we have showed that the modified RDF  should be used instead of the usual RDF *g*(*r*) when computing the density fluctuations in finite volumes for obtaining the local compressibility. However, this does not mean that *g*(*r*) should be replaced by  in all statistical mechanical relations. As an example where the modified RDF should not be used, we mention the contact theorem,^35^ which holds exactly for the HSF and relates the usual RDF at  to the bulk pressure *p* by . In this relation,  cannot be used instead of *g*(σ), since  differs from *g*(*r*) in principle at all points *r*, see Figure 3a. In general, *g*(*r*) should only be replaced by  in expressions involving integrals over the RDF. The infinite volume integral over the RDF is unchanged by the modification.^27^ So, thermodynamic relations involving integrals over the RDF, such as the compressibility equation or the Gibbs adsorption theorem,^35^ are still valid with  instead of *g*(*r*). For relations involving specific points of the RDF, such as the contact theorem, the usual pointlike RDF *g*(*r*) must in general be kept. A second example where the present method should not be used is the calculation of the solvation free energy in a hard sphere fluid with the information theoretical approach.^24,36,37^ In this theory, the probability  for finding exactly zero solvent particles in a volume *V* that equals the size of the solute particle is required. By construction, *P*(*N*) is a discrete probability distribution for *N* particles in *V* and it can be expressed in terms of the fluctuations of the number of particle centers inside the exclusion volume of the solute particle. For computing *P*(*N*), the point-like particle number fluctuations are the natural choice, while the continuous density fluctuations of extended particles introduced here cannot be used directly. The foregoing examples show that our new approach has some limitations. The modified RDF should only be used for volume integrals, and the resulting density fluctuations should not be used to compute quantities corresponding to discrete values of *N*.

## 6 Conclusions

In summary, we have developed a method for calculating density fluctuations ν and the isothermal compressibility κ_T_ at the nanoscale from a modified RDF, which takes account of the finite size of the individual particles. This modification removes the large and systematic  size-dependence of ν that is found with the usual method, i.e., with the particles defined as point-like in the RDF. We have shown that the  term is a nonphysical finite-size effect due to the neglect of the excluded volume. We have applied the new method to HSF in 1D and 3D. We find that at high density, typical for the liquid state, the fluctuations are size-independent down to the smallest physically meaningful sampling volume containing one particle. For the HSF at low density, some size-dependence of ν remains, but it can be made to vanish by using a particle diameter in the RDF, which is smaller than the hard sphere diameter. Simple empirical formulas for the optimum size reduction are given for the HSF in 1D and 3D. The method also gives excellent results for the hard sphere solid. While we have focused on the HSF in this paper, the method can be applied to any system, with a suitable definition of the effective particle size used in the modified RDF. The main findings should be valid for any fluid because they are related to the excluded volume effect, which is independent of the type of particle interactions. The new method has major advantages over the usual method based on point-like particles. First, numerical calculations of particle number fluctuations converge much faster as a function of the sampling volume. For the HSF, a sampling volume containing a single particle gives already well-converged results. Second, density fluctuations can be computed locally with good accuracy using very small sampling volumes, thereby minimizing the finite size effects. This should make it possible to compute the local compressibility and related quantities in inhomogeneous and confined systems, unambiguously and accurately from fluctuation theory. To this end, the RDF needs to be replaced by the general pair distribution function  from which the modified 2-particle density  can be obtained through a multidimensional convolution. However, calculating the two-point function  may be time-consuming. Alternatively, the number fluctuations in a given sampling volume can be computed directly as in the small system method.^5^ For particles that cross the surface of the sampling volume, the volume fraction of the particle inside the volume can be computed analytically as a function of the particle-surface distance to ensure numerical efficiency. Details on the generalization of the present theory to inhomogeneous systems will be given elsewhere.

## Appendix A

### Why Density Fluctuations in a Homogeneous System Should be Size-Independent

Here, we argue that in a homogeneous system, the relative particle number fluctuations ν should be size-independent to be physically meaningful, more precisely: only if ν is size-independent, it can be interpreted as a thermodynamic response function. We consider a homogeneous system at temperature *T* and density , where *N*_0_,. Sampling is done in a finite subvolume *V*. The pressure of the subvolume, obtained by averaging over the  ensemble and over all particles inside *V*, is in principle a function of *V*, *N*_0_, *V*_0_ and *T*, but in a homogeneous system, *p* is obviously independent of *V*, i.e., it is a function of  alone. Assuming that *p* depends on *V* leads to a contradiction. Indeed, when the total system is filled up with small identical subvolumes *V*, the pressure in each subvolume must be the same as the macroscopic pressure, so it cannot be size-dependent. Now, by definition, the pressure function  completely determines the isothermal compressibility as . Since *p* is independent of *V*, so is κ_T_. The foregoing argument clearly shows that the compressibility of an *open* subvolume *V* of a *homogeneous* system is independent of *V*. So, if the compressibility is to be obtained from the fluctuation theory, the density fluctuations must be defined in such a way as to be size-independent. We note that the same reasoning can be done for the variable pair  instead of (). It follows that the derivative of the chemical potential , which is the response function most directly related to the particle number fluctuations in an open system,^19^ is also independent of the subvolume size *V*.
